# An Operation for Pyonephrosis

**Published:** 1883-04

**Authors:** 


### Original Translations and Abstracts.

### AN OPERATION FOR PYONEPHROSIS.

[Concluded from March Number.]

The analysis of this case is interesting, both from an anatomical and from a clinical standpoint. The cause of the renal changes first demands attention. From the history, as given by the patient herself, we could only learn one thing—that in all probability a chronic pyelitis of the left side had existed since early youth. The cause of the accumulation of urine we recognise only by combining the clinical history and the autopsical appearances.

In no part of the urinary canal, from urethra to the origin of the ureter from the pelvis, could any change in the lumen of the tube be found, neither narrowing nor dilatation.

The insertion of the ureter in the anterior wall of the pelvis of the kidney above its deepest part, together with the course which it apparently ran in the anterior wall of the pelvis—these we hold responsible for the accumulation of urine in the left kidney.

From such an arrangement of the parts, the pressure of the fluid that collected in the kidney must have narrowed the insertion of the ureter, and occluded it more and more with increasing pressure.

I fancy that this "*retention arrangement*" in the renal pelvis was produced in a manner similar to that occurring in the urinary bladder, independent of prostatic hypertrophy. Thus, if the elasticity of the inner coat of the bladder be impaired, either by chronic catarrh or by senile changes, the power to force the urine into the urethra becomes insufficient. In this way the reservoir is incompletely emptied, and hence enlarges. If this enlargement be partial, having a sac-like form behind the vesical urethral opening, so much of the hydrostatic pressure as now acts upon that part of the urethra which is prominent in the bladder, induces compression of the urethra's lumen, and in this way works against the component pressure exerted on the vesical wall, which pressure, normally, should enlarge the ostium urethræ. If both pressures be equal no urine can leave the bladder. Transferring similar conditions to the renal pelvis, we readily see how a pyelitis that existed from youth could lead to an enlargement of the pelvis by diminishing the elasticity of its walls. If the pelvis fills behind the ureter's insertion, the latter will be steadily pushed forward, until it finally lies in the anterior wall of the pelvis. And if the process continues, the anterior wall will be so closely approximated to the posterior wall of the ureter that the latter will seem to run its course far inside the anterior pelvic wall.

If such a ureter be cut longitudinally, it will look as if its posterior wall extended, as a valve, fully into the lumen of the renal pelvis.

When the ureter takes this course its posterior wall is subject to the pressure of the fluid in the pelvis, and thus the lumen of the ureter is occluded.

This state of things leads, primarily, to a diminished flow, and subsequently to complete retention. The correctness of my idea that there existed an enlargement of the pelvis rather than any anomaly of insertion of the ureter, was proven by finding an enlarged right pelvis without any trace of mechanical obstruction to the flow.

Of special practical importance was the enormous distension of the calyx compared to the moderate dilatation of the renal pelvis. In consequence of this state of affairs a calyx was opened instead of the pelvis.

To have struck the latter the incision should have been made to the inner side of the descending colon, *i.e.*, should have been carried further the linea alba than my incision. This shows that—apart from floating kidneys—there are cases of pyonephrosis where one is forced (contrary to the prevailing opinion) to make an intra-peritoneal incision into the organ.

That this can be successfully accomplished, and that, too, without first artificially uniting the cyst with the abdominal wall, is proven by this case, wherein not even a trace of peritonitis was discoverable.

Although our aim is, to be sure, the opening of the pelvis, yet an incision into a calyx may also lead to the desired result if the opening communicating with the pelvis be sufficiently large, or if it can subsequently be made so by dilatation by means of the thermocautery knife.

Another point of the highest importance, and upon which the prognosis of most of these cases depends, is the condition of the fellow kidney. Here we found grave lesions in the right kidney that were not diagnosticated during life, because of the *normal quantity of urine*, absence of cardiac hypertrophy, and because of the strong and well-nourished state of our patient.

Perhaps an examination of the urine for the *amount of urea* would have given us some intimation of it.

This fact must serve as a never-to-be-forgotten warning to us not to attempt, as a rule, extirpation for hydro- or pyo-nephrosis, even though the urine be sufficient in quantity; for the loss of the smallest portion of parenchyma capable of secretion may lead to a fatal uræmia, if the fellow kidney be not able to properly perform its function.

We must mention as a fact of great theoretical as well as practical interest the absence of any cardiac hypertrophy in spite of disease of both kidneys, which must undoubtedly have led to as great an occlusion of the tubular structure as would have been caused by advanced granular atrophy of both organs.

In spite of this, however, the amount of urine was uninfluenced by absence of hypertrophy of the heart.

Now, when we add to this that the ordinary conditions for cardiac hypertrophy were present, viz., a healthy and vigorous constitutional condition and an early development of the disease, we regard these facts as anything but favorable to the "*mechanical theory*" of Traube, according to which renal vascular changes lead to increased arterial pressure, and hence to cardiac hypertrophy.

The probability is, that the disproportion between the cardiac muscular power and the resistance in the vascular system of the kidneys was responsible for the fatal ending in our case.

Evidently the heart had preserved but a labile equilibrium prior to the moment when, the cardiac muscle refusing to do its work, the arterial tension sank to a minimum and the pulse-rate rose rapidly.

Heart-failure was the immediate cause of the uræmia.

What element was it that so suddenly and direfully annulled the heart-functions on the night between November 3d and 4th, when all had been normal (with reference to that organ) during the evening of the 3d?

I believe that the morphia injections given that night may reasonably be held accountable therefor.

Even if there were other weakening elements, such as four weeks of fever, prolonged use of chloroform as a narcotic and imperfect digestion of the food, still the malady only assumed a fatal form after the hypodermatics of morphine. The action of this drug was the last drop that caused the bucket to overflow.

These, then, are the practical deductions from the case in question:

I. In a case of hydro- or pyo-nephrosis of *one* side, even if the secretion be normal and there be no cardiac hypertrophy, we must always be prepared to meet disease of the fellow kidney.

II. Hence the formation of a fistulous communication between the pelvis of the kidney and the external world is preferable to extirpation when renal disease has been diagnosticated.

III. Should cystic enlargement of a calyx be more prominent than the pelvic dilatation, we may be compelled to operate, even though the peritoneum be wounded. And this operation can be successfully performed without first artificially uniting the tumor with the abdominal walls.

IV. The use of narcotics must be restricted, on account of their weakening the heart power—in all forms of renal disease where considerable resistance is offered to the circulatory current.—*Berl. Kl in Wochen,* No. 51 : 1882.

---

A correspondent of the *British Medical Journal* (Jan. 13, p. 90) states that he has found the application of a strong solution of chromic acid, three or four times, by means of a camel's hair pencil, to be the most efficient and easy method of removing warts. They become black and soon fall off.

